# Integrating the Porosity/Binder Index and Machine Learning Approaches for Simulating the Strength and Stiffness of Cemented Soil

**DOI:** 10.3390/ma18245504

**Published:** 2025-12-07

**Authors:** Jair De Jesús Arrieta Baldovino, Oscar E. Coronado-Hernandez, Yamid E. Nuñez de la Rosa

**Affiliations:** 1 Department of Civil Engineering, Universidad de Cartagena, Cartagena de Indias 130015, Colombia; 2 Instituto de Hidráulica y Saneamiento Ambiental, Universidad de Cartagena, Cartagena de Indias 130001, Colombia; ocoronadoh@unicartagena.edu.co; 3 Faculty of Engineering and Basic Sciences, Fundación Universitaria Los Libertadores, Bogotá 110231, Colombia

**Keywords:** cemented soils, crushed aggregate residue (CAR), porosity–binder index, machine learning, unconfined compressive strength, small-strain shear modulus, sustainable geomaterials

## Abstract

This study evaluates the mechanical performance and predictive modeling of fine-grained soils stabilized with crushed aggregate residue (CAR) or crushed limestone waste (CLW) and Portland cement by integrating the porosity–binder index ($\eta /C_{iv}$) and Machine Learning (ML) techniques. Laboratory testing included unconfined compressive strength (q_u_) and small-strain shear modulus (*G_o_*) measurements on mixtures containing 15% and 30% CAR and 3% and 6% cement, compacted at dry unit weights between 1.69 and 1.81 g·cm^−3^ and cured for 7 and 28 days. Results revealed that strength and stiffness increased significantly with both cement and CAR contents. The mixture with 30% CAR and 6% cement exhibited the highest mechanical performance at 28 days (q_u_ = 1550 kPa and *G_o_* = 6790 MPa). When mixtures are compared within the same curing period, the role of CAR and cement becomes evident. At 28 days, increasing CAR from 15% to 30% led to a moderate rise in q_u_ (from 1390 to 1550 kPa) and *G_o_* (from 6220 to 6790 MPa). Likewise, at 7 days, increasing cement from 3% to 6% at 15% CAR produced significant gains in q_u_ (207 to 693 kPa) and *G_o_* (2090 to 4120 MPa). The porosity–binder index showed strong correlations with q_u_ (*R*^2^ = 0.94) and *G_o_* (*R*^2^ = 0.92). The ML models further improved accuracy, achieving *R*^2^ values of 0.99 for q_u_ and 0.97 for *G_o_*. Although the index already performed well, the additional gain provided by ML is meaningful because it reduces prediction errors and better captures nonlinear interactions among mixture variables. This results in more reliable estimates for mix design, confirming that the combined use of $\eta /C_{iv}$ and ML offers a robust framework for predicting the behavior of soil–cement–CAR mixtures.

## 1. Introduction

Soil stabilization is the controlled modification of the physical and mechanical properties of soil—such as texture, structure, strength, permeability, volumetric stability, and durability—through mechanical or chemical techniques that accelerate the construction process and ensure the stability and safety conditions required for engineering works [1]. It is uncommon to find a fine-grained soil with expansive behavior that can be directly used as a construction material capable of supporting loads without improving some of its properties. Moreover, most construction techniques involve some degree of compaction to produce strong and stable material. The primary purpose of stabilization is to enhance the engineering properties of the soil until it becomes suitable for construction activities; that is, to ensure it is sufficiently durable, does not undergo excessive swelling or shrinkage, does not deform to an extent that may affect the structure, and remains stable [2].

Stabilization techniques are employed when the soil at a construction site exhibits low mechanical strength, is inefficient in transmitting structural loads, or presents characteristics that could hinder the technical, economic, or social feasibility of an engineering project. Since the soil serves as the foundation for virtually all civil works—such as roads, tunnels, structures, and buildings- it is crucial to apply suitable stabilization methods to ensure its strength and durability [3].

The application of stabilization techniques is recommended once it has been identified that the in situ soil lacks the mechanical capacity to provide adequate structural stability [4]. These methods are widely adopted in the construction field, as they allow for lower material costs and yield satisfactory outcomes in the implementation of civil works. The most common stabilizing agents used to enhance the mechanical behavior of soils are cement and lime; however, their production processes are costly and result in high carbon dioxide (CO_2_) emissions, significantly contributing to environmental pollution [5]. Consequently, there is a growing need to employ alternative stabilizing agents that are both environmentally friendly and economically feasible.

Previous studies in Cartagena (Colombia) have evaluated the improvement of soil mechanical properties through chemical stabilization with lime; the mechanical behavior of low-plasticity clayey soils stabilized with cement; and the stabilization of granular and subgrade soils using oily sludge [6]. All reported positive outcomes in soil performance after stabilization. Similarly, the optimal stabilizing agent was investigated for a sandy–clayey soil contaminated with gasoline and treated with lime, cement, and asphalt; cement exhibited the best performance in terms of cohesion and shear strength parameters [7].

Crushed aggregate residue (CAR) from limestone has also been investigated as an alternative binder for soil stabilization. Studies have shown that when incorporated powdered rocks into fine-grained soils, it can improve soil strength (unconfined compressive, CBR and resilient modulus Mr) and reduce both the liquid limit and plasticity index. Furthermore, the inclusion of rock waste as a stabilizing agent contributes to decreasing the deformability of weak clayey soils [8,9]. Thus, Table 1 presents the influence of some type of powdered rocks in the strengthening of soils and some geomaterials as reclaimed asphalt pavement.

As shown in Table 1, Consoli et al. [10] investigated the dynamic modulus of reclaimed asphalt pavement (RAP) blended with powdered rock and Portland cement to promote sustainable pavement recycling. Mixtures with a 70:30 RAP-to-rock ratio and 3–7% cement were tested at different dry unit weights (20–22 kN/m^3^) through unconfined compressive and indirect tensile strength tests. Results showed that increasing cement content and compaction improved mechanical strength, and that the porosity-to-cement ratio effectively normalized the behavior of the mixtures, yielding strong correlations (*R*^2^ = 0.95). The study demonstrated that this ratio could guide the selection of optimal cement content and porosity for cost-effective and high-performance pavement base materials.

Blayi et al. [11] examined the effect of rock powder on the geotechnical behavior of expansive soils to assess improvements in their engineering properties. Laboratory tests—including standard compaction, free swell, unconfined compressive strength (q_u_), California Bearing Ratio (CBR), and permeability—were performed on high-plasticity silt mixed with 0–40% rock powder and cured for up to 28 days. The addition of rock powder reduced the liquid limit, plastic limit, and plasticity index by up to 40%, while q_u_ and CBR increased by 98.1% and 337.1%, respectively, at 24% replacement. Direct shear tests showed a 36.7% reduction in cohesion and a 95.3% increase in internal friction angle. The study concluded that approximately 24% of rock powder content provides the most effective stabilization for expansive soils.

Gidigasu et al. [12] investigated the use of waste crushed rock and spent carbide blends for stabilizing lateritic soils as a sustainable road base material. The study aimed to evaluate the combined influence of these waste materials on the strength and engineering performance of weak lateritic soils as eco-friendly alternatives to conventional stabilizers. Laboratory tests—including particle size distribution, Atterberg limits, specific gravity, compaction, and California Bearing Ratio (CBR)—were conducted to assess improvements. The results showed that adding 20% crushed rock waste reduced the fine content by about 31% and decreased the plastic limit and plasticity index by 35–40%. Increasing the spent carbide content raised the fine fraction but remained within the recommended classification for road base materials in Ghana for up to 7% spent carbide, which also yielded significant CBR improvements. The optimal stabilization condition was achieved with 40% crushed rock waste and 7% spent carbide, providing a balanced enhancement of the geotechnical properties for use in road base applications.

Finally from the analysis of Table 1, Pasche et al. [13] investigated the effect of polypropylene fiber reinforcement on the mechanical behavior of reclaimed asphalt pavement (RAP)–powdered rock (PR)–Portland cement (PC) mixtures. The study evaluated resilient modulus, indirect tensile strength, and durability, correlating the results with the porosity-to-cement ratio to establish a rational mix design for both fiber-reinforced and unreinforced blends. Specimens were prepared with a 70/30 RAP–PR ratio, 3–7% cement, 0.5% fiber, and dry unit weights of 20–22 kN/m^3^ at 9% optimum moisture content. Results indicated that unreinforced cemented mixtures achieved higher resilient modules, tensile strength, and durability than fiber-reinforced ones, with the latter showing up to 40% lower mechanical performance. The porosity-to-cement index proved effective in predicting resilient modulus (*R*^2^ = 0.90). The authors concluded that 24 mm polypropylene fibers were unsuitable for improving mechanical behavior due to their geometry and interaction with the granular structure of the mixtures.

The use of Machine Learning (ML) has gained increasing attention from researchers in the field of soil stabilization and improvement in recent studies [14]. In this context, ML models have demonstrated superior predictive capability compared with conventional empirical geotechnical formulas, owing to their more complex structures [15,16]. Zhang et al. [17] interpreted the relationships between cement content, water content, curing age, and fine-grained fraction with the compressive strength by employing a dataset of 566 samples. Ikramul et al. [18] used ML models to investigate the unconfined compressive strength behavior of stabilized soft soils reinforced with polypropylene columns.

The porosity–binder index (*η*/B_iv_) is a dimensionless parameter proposed to rationalize the relationship between soil structure and binder content in cemented materials. It integrates the void ratio or porosity (*η*) with the volumetric binder content (B_iv_), providing a unified framework that captures the combined influence of compaction and cement dosage on mechanical performance. Previous studies have shown strong correlations between this index and key parameters such as unconfined compressive strength, stiffness, and tensile resistance in artificially cemented soils [6,19,20].

Although both the porosity–binder index and machine learning models have independently shown strong predictive capabilities in stabilized soils, no previous study has integrated these two approaches to evaluate the mechanical behavior of soil–cement mixtures incorporating crushed aggregate residue (CAR). The novelty of this work lies in combining a mechanistic, physically grounded index (*η*/C_iv_) with a data-driven ML framework to simultaneously interpret, model, and enhance prediction accuracy for q_u_ and *G_o_*. This dual methodology provides new insight into how binder content, porosity, curing, and CAR fraction interact, and offers a more robust and comprehensive tool for designing sustainable geomaterials

Therefore, this study aims to investigate the prediction of unconfined compressive strength (q_u_) and small-strain shear modulus (*G_o_*) of fine-grained soils stabilized with crushed aggregate residue (CAR) and Portland cement by integrating the porosity–binder index and Machine Learning (ML) techniques. Experimental tests were conducted to evaluate the influence of CAR and cement contents, curing time, and dry unit weight on the mechanical performance of the mixtures. The porosity–binder index was used as a rational framework to normalize the effects of mixture design variables, while ML algorithms were applied to develop predictive models capable of estimating q_u_ and *G_o_* with high accuracy. This integrated approach provides new insights into the behavior of cemented granular–fines mixtures and contributes to the advancement of sustainable soil stabilization practices through data-driven modeling.

### Objective and Scope

The objective of this study is to evaluate the mechanical behavior and predictive modeling of fine-grained soils stabilized with crushed aggregate residue (CAR) and Portland cement through the combined use of the porosity–binder index and machine learning (ML) techniques. The scope includes: (i) conducting laboratory tests to quantify the influence of CAR content, cement dosage, compaction energy, and curing time on unconfined compressive strength (q_u_) and small-strain shear modulus (*G*_o_); (ii) establishing empirical power-law relationships between mechanical response and the porosity–binder index; and (iii) developing and validating ML models to assess their predictive performance relative to the empirical framework. The study is limited to controlled laboratory conditions, Type III Portland cement, CAR sourced from a single quarry, and curing ages of 7 and 28 days.

## 2. Materials and Methods

**Figure 1:**
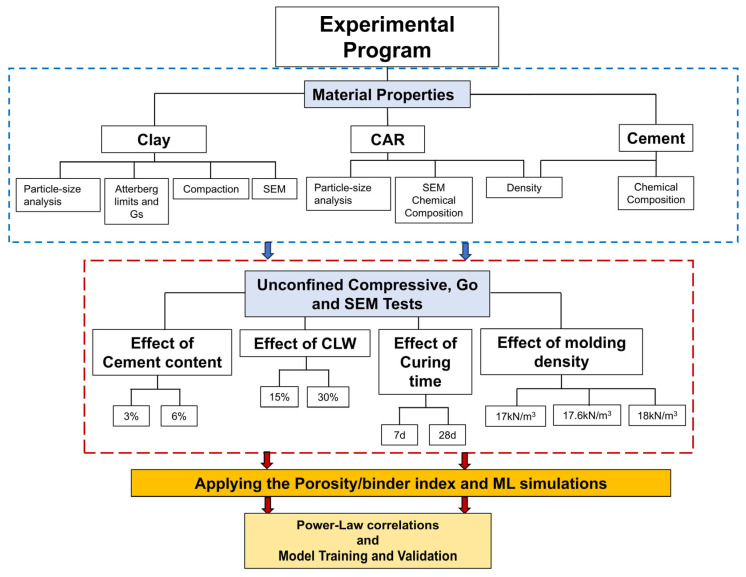
Experimental Program Flowchart.

The research program encompassed the characterization of the constituent materials (clay, Ordinary Portland cement, and crushed aggregate residue), specimen molding and curing, unconfined compressive strength (q_u_) assessment, determination of initial shear stiffness (*G_o_*), and microstructural investigation through SEM-EDX techniques (Zeiss, model EVO MA 15, Carl Zeiss Ltd., Cambridge, UK). The expimental program flowchart is presented By Figure 1.

### 2.1. Materials

In the northern expansion sector of Cartagena de Indias (Colombia), clayey soils were retrieved from a natural slope, as reported by Baldovino et al. [21]. A detailed program of physical, chemical, and mineralogical characterization was carried out. Grain-size distribution was obtained through laser diffraction and gradding using a hexametaphosphate dispersing solution [17]. The liquid and plastic limits were defined following ASTM D4318 [22], while the specific gravity of solids was assessed according to ASTM D854 [23]. Compaction properties were examined by means of the standard Proctor procedure (ASTM D698 [24]), and the classification was subsequently established under ASTM D2487 [25], which placed the material within the low-plasticity clay (CL) group.

**Figure 2:**
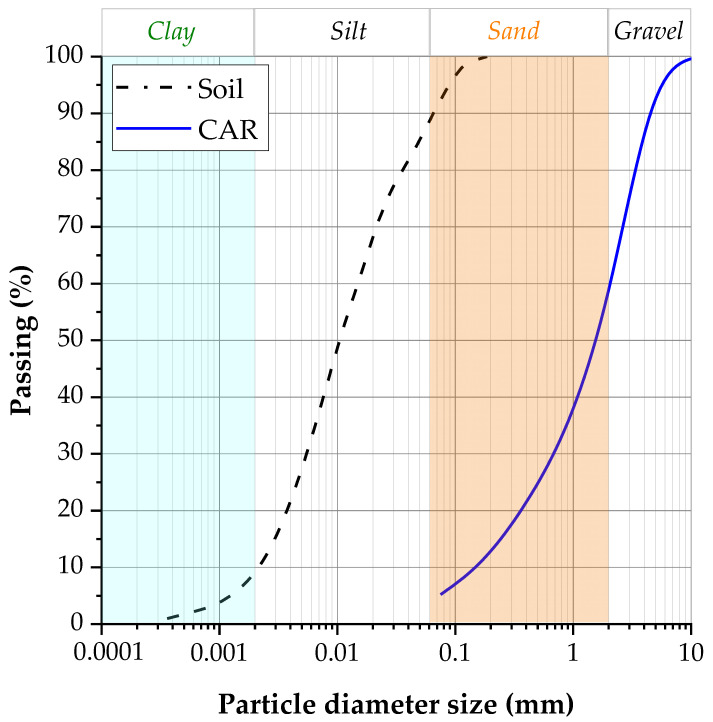
The granulometric curve of the soil sample and crushed limestone waste (CAR).

The particle-size curve (Figure 2) revealed that the soil contained 12% fine sand, 78% silt, and 10% clay, results that are consistent with the Massachusetts Institute of Technology classification scheme. Geotechnical index testing yielded a liquid limit of 42% and a plastic limit of 26.05%, producing a plasticity index of 15.95%. The coefficients of uniformity (C_u_) and curvature (C_c_) were calculated as 7.14 and 6.9, respectively. The specific gravity of particles averaged 2.80 g·cm^−3^.

Chemical and mineralogical features of soil were investigated using an Oxford Penta Field-Effect Transistor (FET125) Precision X-ACT spectrometer (Oxford Instruments, Abingdon, UK), complemented with a micro mass analyzer LAMMA (Light Anion Microprobe Mass Analyzer)-1000 (Leybold-Heraeus GmbH, Cologne, Germany) and energy-dispersive X-ray analysis. The results, summarized in Table 2, highlighted silicon and aluminum as the dominant constituents of soil. X-ray diffraction confirmed the presence of highly crystalline phases, mainly kaolinite, quartz, and muscovite. The compaction study determined an optimum moisture content of 18% and a maximum dry unit weight of 17.60 kN·m^−3^.

Crushed aggregate residue (CAR) or crushed limestone waste CLW was obtained from a quarry located near Cartagena de Indias, and its original particle size distribution was preserved. A comprehensive characterization of the material was performed, including chemical composition, particle size distribution, and density analyses. The grain-size distribution curve is illustrated in Figure 2, while the physical properties and chemical composition are summarized in Table 2 and Table 3, respectively. CAR presents high percentage of CaO. The material exhibits a well-graded coarse distribution (*D*_50_ = 1.6 mm) with high angularity and rough surface texture, which enhances mechanical interlock when blended with fine-grained soils. Its granulometric structure comprises gravel- and sand-sized particles (41% and 32%, respectively), contributing to a denser packing and reduced porosity when incorporated into the soil–cement mixture [26].

**Figure 3:**
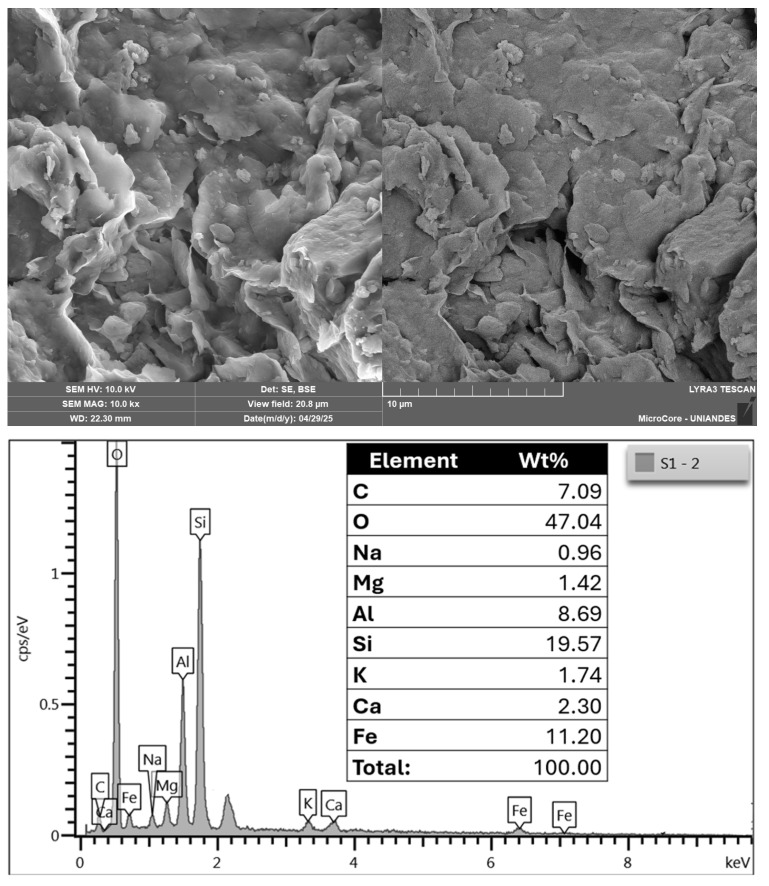
SEM images and EDX microanalysis of the soil sample.

Figure 3 presents the microstructural features of soil obtained through scanning electron microscopy–energy dispersive X-ray spectroscopy (SEM-EDX), and Figure 3 also displays its elemental composition determined by SEM–EDX microanalysis of soil. In Figure 3, the SEM image at 10,000× magnification reveals angular and irregular particles with rough surface textures and localized porosity.

**Figure 4:**
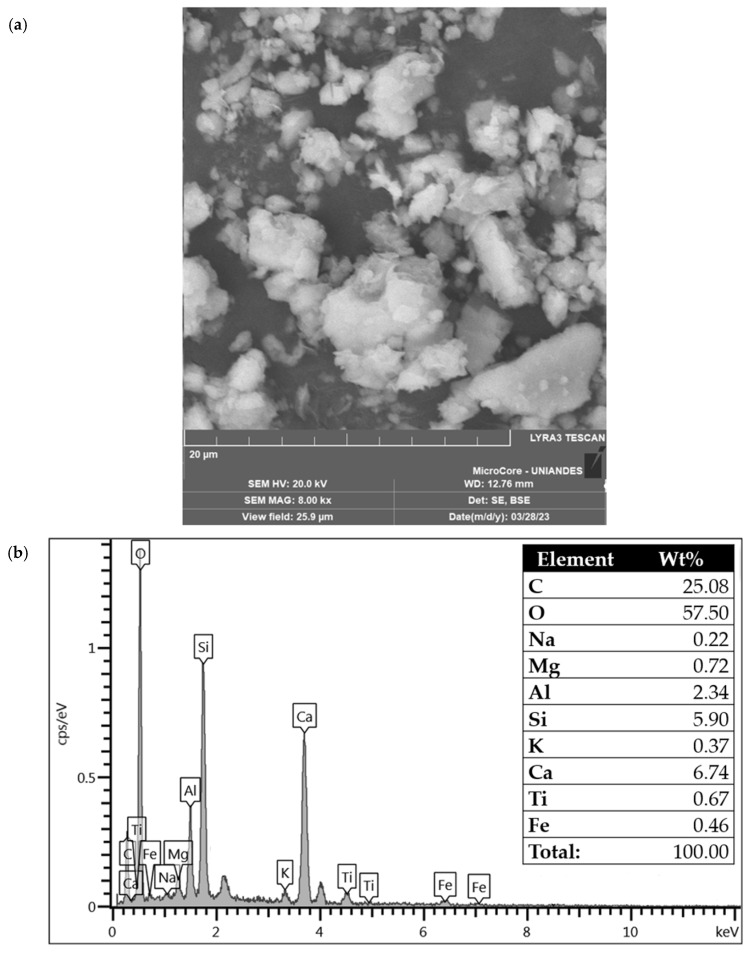
SEM-EDX of CAR. (**a**) SEM of CAR magnified at 20,000 times; (**b**) EDX chemical analysis of CAR detected region.

Figure 4 presents the SEM-EDX analysis of CAR. The CAR presents angularity and high calcium and silicon content from the CaCO_3_ of the calcite of the crushed limestone rock. Such morphological characteristics favor an effective mechanical interlock and bonding between the CAR particles and the soil–cement matrix.

A Type III Portland cement, characterized by its high early strength, was employed as the primary binder. Its chemical composition was dominated by calcium oxide (60.7%), magnesium oxide (4.1%), and sulfur trioxide (3.0%). According to the manufacturer’s data, the cement exhibited an insoluble residue of 0.77% and a fineness of 0.04%. After 28 days of curing, its compressive strength reached 53 MPa. The specific gravity of the cement particles, determined in accordance with ASTM C150 [27], was 3.11 g·cm^−3^. This binder was selected due to the extensive production and availability of Portland cement in northern Colombia, where abundant limestone resources and local manufacturing facilities exist. Distilled water was utilized for all testing and specimen preparation processes.

### 2.2. Experimental Program

The experimental program, illustrated in the flowchart of Figure 4, was divided into two main stages: (i) raw materials characterization and (ii) mechanical and microstructural evaluation.

In the first stage, the physical and chemical properties of the three primary components—soil clay, crushed aggregate residue (CAR), and Ordynary Portland cement—were determined. For plastic clay, tests included particle-size distribution, Atterberg limits, specific gravity, standard compaction, and scanning electron microscopy (SEM) imaging. CAR characterization comprised particle-size analysis, density measurement, and microstructural and chemical composition analyses using SEM–EDX. The Portland cement was also characterized in terms of density and chemical composition.

The second stage involved the preparation and testing of soil–CAR–cement mixtures to investigate the influence of key variables—cement content, CAR content, curing time, and molding density—on strength and stiffness behavior. Unconfined compressive strength (q_u_), small-strain stiffness (*G_o_*), and SEM-EDX analyses were conducted on specimens compacted at three dry unit weights (17, 17.6, and 18 kN·m^−3^). The cement and CAR contents were varied between 3–6% and 15–30%, respectively, with curing periods of 7 and 28 days. The compaction conditions were established based on standard Proctor tests, which yielded a maximum dry unit weight of 17.6 kN·m^−3^ and an optimum moisture content of 18%. These parameters were used to select two compaction levels: one below and one above the optimum condition.

**Figure 5:**
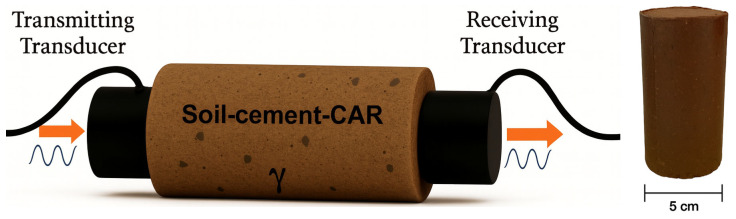
Stiffness and unconfined compressive specimens.

Cylindrical specimens with dimensions of 50 mm in diameter and 100 mm in height were prepared in triplicate for each mix (Figure 5). The preparation process included controlled weighing, mixing, compaction, sealing, and curing under constant temperature (27 °C) and relative humidity (95%). All specimens met dimensional and moisture tolerances within ±2% of the target values, following ASTM D1633 [28] standards. After curing, samples were immersed in water for 24 h to minimize suction effects prior to ultrasonic pulse velocity and q_u_ testing, conducted according to ASTM C597 [29] and ASTM D1633 [28], respectively. Test specimen and stiffness approach test is presented in Figure 5.

The relationship between q_u_ and the porosity-to-cement volumetric index ($\eta /C_{iv}^{x}$) was then evaluated, as expressed in equation: $q_{u}=A{\left(\eta /C_{iv}^{x}\right)}^{-B}$. This approach enables normalization of the strength response, considering both the degree of compaction and the cement volumetric content. In this study, the CAR fraction was explicitly incorporated into the porosity computation, representing an innovation compared to previous formulations that considered other waste-based binders such as lime sludge, sugarcane bagasse ash, carbide lime, or glass powder. The constants A, B, and *x* were calibrated to describe the mechanical response of the stabilized mixtures, as previously proposed by Consoli et al. [29] and refined by Arrieta et al. [30].

Finally, SEM–EDX microanalyses were performed on representative specimens (Soil sample 1= 3%C + 15%CAR and Soil sample 2 = 6%C + 30%CAR) after 28 days of curing to identify microstructural changes and cementitious reaction products. The specimens were polished using ethanol, vacuum-dried, gold-coated, and analyzed under magnifications of up to 10,000× using a LIRA3 TESCAN microscope (Universidad de los Andes, Bogotá, Colombia).

### 2.3. ML Methodology

**Figure 6:**
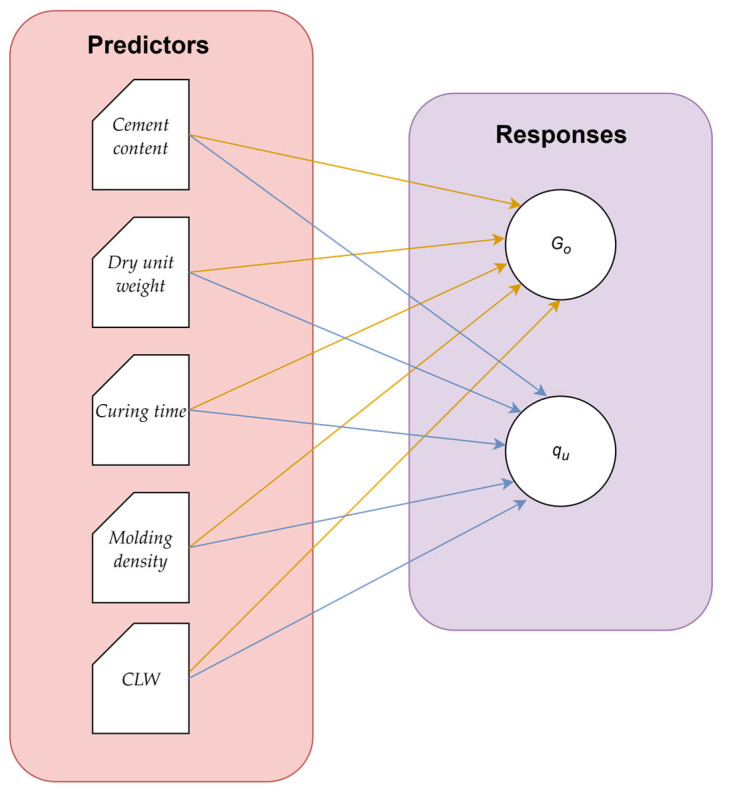
Relationships between predictors and responses for ML modeling.

To further advance the prediction of *G_o_* and q_u_ values, machine learning (ML) algorithms were applied. In this context, during the experimental program, cement content, dry unit weight, curing time, molding density, and CAR were used as input variables for predicting the aforementioned responses. Figure 6 illustrates the relationships between the predictors and the responses considered in this study, where these five input features were employed to identify and predict the two target variables.

**Figure 7:**
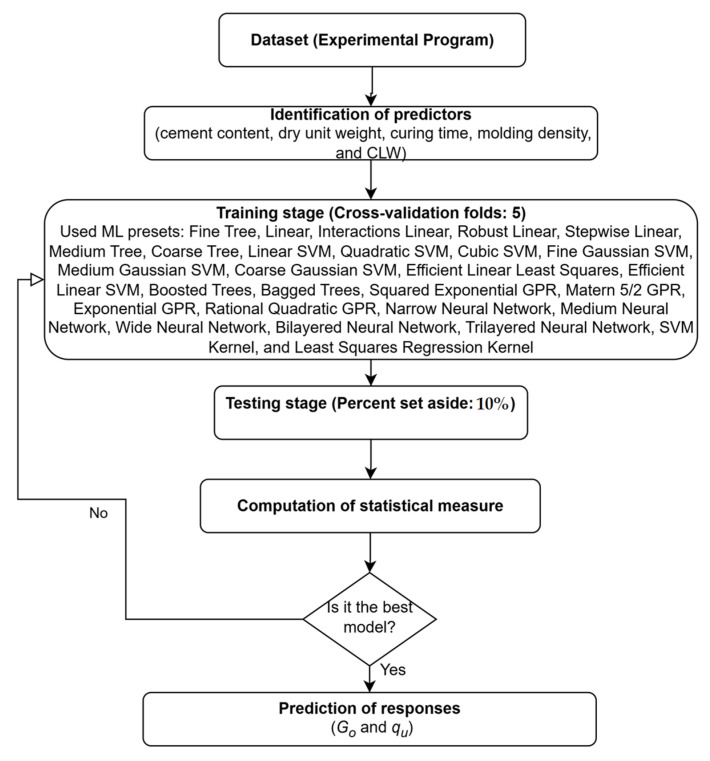
ML methodology flow diagram.

Figure 7 presents the methodology employed in this research for computing the response variables (*G*_o_ and q_u_). The calculations were performed using MATLAB R2024b, specifically through the Regression Learner App, which facilitated the training, validation, and performance assessment of the machine learning models.

In order to identify the most suitable machine learning (ML) preset, several techniques were evaluated using a comprehensive set of algorithms, including decision trees, linear regression, support vector machines (SVM), Gaussian Process Regression (GPR), ensemble methods, neural networks, and kernel-based models. A total of 28 ML algorithms were tested.

Initially, the algorithms were trained using a 5-fold cross-validation scheme to ensure robust model generalization. Subsequently, during the testing stage, 10% of the dataset was set aside to verify that the fitted models did not exhibit overfitting issues.

The statistical metrics employed to assess and compare model performance for the two target responses (*G_o_* and q_u_) were the coefficient of determination (R^2^) and the root mean square error (RMSE). These indicators are defined as follows:

(1) $$RMSE=\sqrt{\frac{1}{N}\sum_{i=1}^{N}{\left(T_{i}-P_{i}\right)}^{2}}$$

(2) $$R^{2}=1-\frac{\sum_{i=1}^{N}{\left(T_{i}-P_{i}\right)}^{2}}{\sum_{i=1}^{N}{\left(T_{i}-\bar{T}\right)}^{2}}$$

where N corresponds to the 72 data points collected during the experimental program, $T_{i}$ is the true value (obtained from the experiments), $P_{i}$ is the value predicted by the ML model, and $\bar{T}$ represents the mean value of each response (*G_o_* or q_u_) from the experimental dataset.

In this research, the Gaussian Process Regression (GPR) demonstrated the best fit during the validation and testing stage. It is based on a probabilistic model that follows a Gaussian distribution. It is yielding by [31]:

(3) $$y=x^{T}\beta +\varepsilon$$

where y = response of the model (G_o_ or q_u_), x = predictor variables (cement content, dry unit weight, curing time, molding density, and CAR), ε = error variance, and β = coefficients estimated from the experimental program.

During the training stage, the following kernel function were evaluated [32]:

- Square exponential kernel

(4) $$k(\left.x_{i},\;x_{j}\right|\theta )=\sigma_{f}^{2}\exp\left[-\frac{1}{2}\frac{{\left(x_{i}-x_{j}\right)}^{T}(x_{i}-x_{j})}{\sigma_{l}}\right]$$

where $x_{i}$ = predictor value, $k(x_{i},x_{j})$ = covariance function, θ = kernel parameters, $\sigma_{l}$ = characteristic length scale, $\sigma_{f}$ = signal standard deviation.

- Exponential kernel

(5) $$k(\left.x_{i},\;x_{j}\right|\theta )=\sigma_{f}^{2}\exp\left(-\frac{r}{\sigma_{l}}\right)$$

where r = Euclidean distance between points $x_{i}$ and $x_{j}$.

- Matern 5/2

(6) $$k(x_{i},\;x_{j})=\sigma_{f}^{2}\left(1+\frac{\sqrt{5}r}{\sigma_{l}}+\frac{5r^{2}}{3\sigma_{l}^{2}}\right)\exp\left(-\frac{\sqrt{5}r}{\sigma_{l}}\right)$$

- Rational quadratic:

(7) $$k(\left.x_{i},\;x_{j}\right|\theta )=\sigma_{f}^{2}{\left(1+\frac{r^{2}}{2\alpha \sigma_{l}^{2}}\right)}^{-\alpha }$$

where α = positive-valued scale-mixture parameter.

## 3. Results and Discussion

### 3.1. Effects of Porosity/Cement Index and Curing Periods on Strength and Stiffness for Soil−CAR−Cement Blends

**Figure 8:**
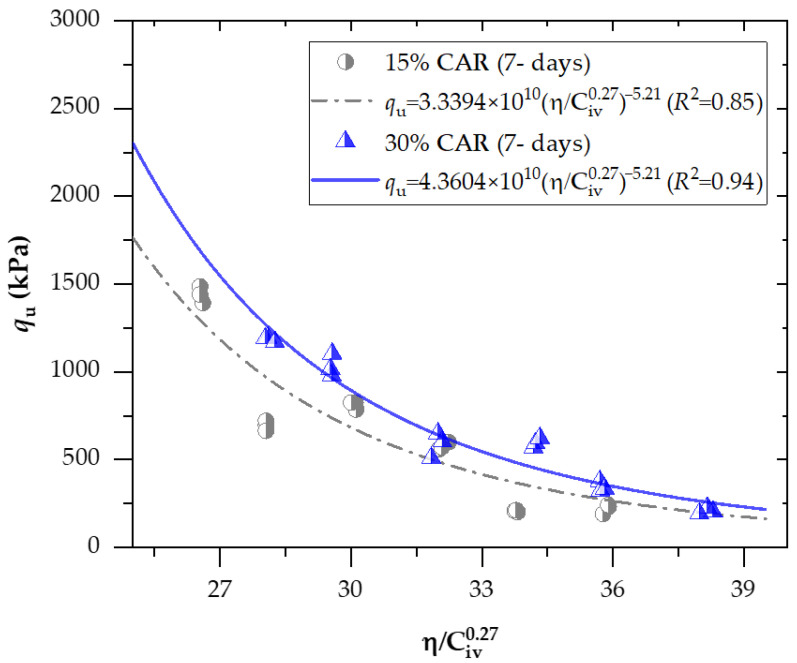
Effects of the porosity-to-binder index (adjusted to *x* = 0.27) on the unconfined compressive strength of soil−CAR−cement compacted blends, considering 7 days of curing.

**Figure 9:**
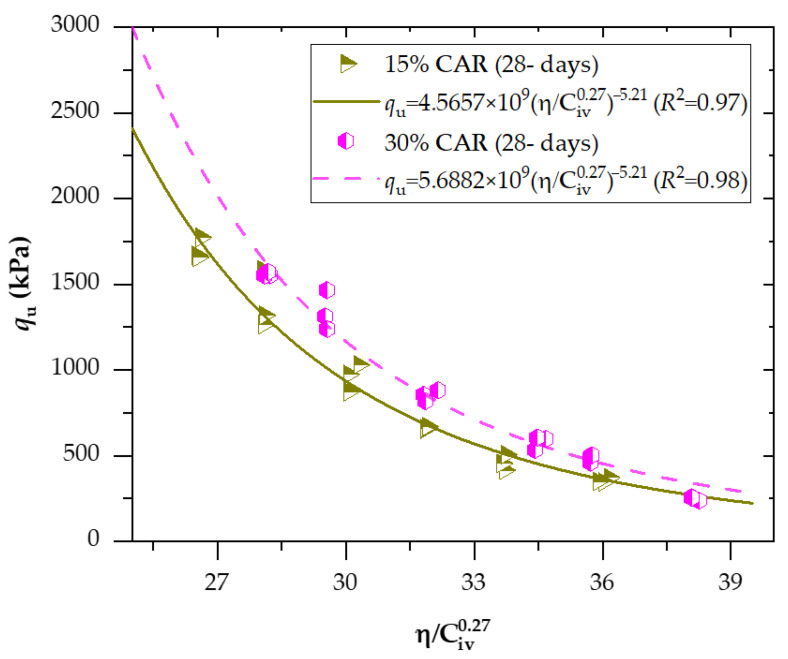
Effects of the porosity-to-binder index (adjusted to *x* = 0.27) on the unconfined compressive strength of soil−CAR−cement compacted blends, considering 28 days of curing.

**Figure 10:**
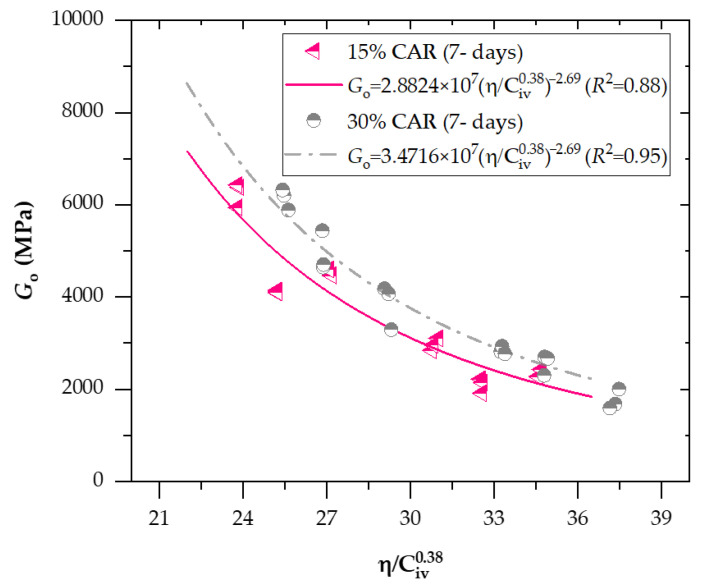
Effects of the porosity-to-binder index (adjusted to *x* = 0.38) on the unconfined compressive strength of soil-CAR-cement compacted blends, considering 7 days of curing.

**Figure 11:**
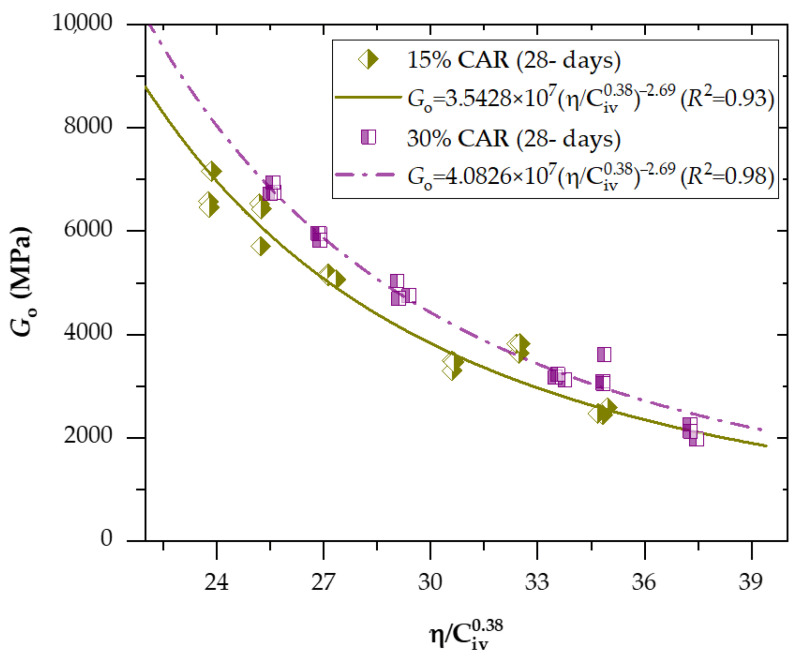
Effects of the porosity-to-binder index (adjusted to *x* = 0.38) on the stiffness of soil−CAR−cement compacted blends, considering 28 days of curing.

Figure 8 and Figure 9 present the effects of the porosity-to-binder index (adjusted to an exponent of *x* = 0.27) on the unconfined compressive strength of soil−CAR−cement compacted blends, considering 7 and 28 days of curing, respectively. In addition, Figure 10 and Figure 11 exhibit the effects of the porosity-to-binder index (adjusted to an exponent of *x* = 0.38) on the stiffness *G*_o_ of soil−CAR−cement compacted blends, considering 7 and 28 days of curing, respectively.

The exponential regression curves (Figure 8 and Figure 9) were developed from a dataset of 72 experimental samples, corresponding to triplicate testing of each mixture condition. Dataset adequacy was confirmed through low variability among replicates, high goodness-of-fit, and unbiased residual distributions across the porosity–binder range, indicating that the sample size and statistical consistency were sufficient for robust regression modeling.

The results clearly show that cement content and porosity were the most influential parameters controlling both strength and stiffness. Increasing cement from 3% to 6% significantly enhanced the unconfined compressive strength (q_u_) and small-strain shear modulus (*G*_o_) of all mixtures. For example, at 15% CAR and 7 days, q_u_ rose from 207 kPa to 693 kPa, a 234% improvement, while *G*_o_ increased from 2.09 GPa to 4.12 GPa. After 28 days, the mixture with 6% cement achieved 1.39 MPa (q_u_) and 6.22 GPa (*G*_o_), compared to only 0.46 MPa and 3.76 GPa for the 3% cement mixture. Similar behavior was observed at 30% CAR, where q_u_ increased from 0.48 MPa to 1.33 MPa and *Go* from 3.24 GPa to 5.90 GPa as cement content rose. These results confirm that higher cement dosage promotes stronger cementitious bonding through the formation of C–S–H and C–A–S–H gels, improving the interparticle connections and the overall rigidity of the stabilized matrix.

The addition of crushed aggregate residue (CAR) also positively influenced the mechanical performance of the mixtures by improving particle packing and creating a more stable skeletal structure. When the CAR content was increased from 15% to 30%, both q_u_ and *G*_o_ showed noticeable improvements, particularly at higher cement dosages. For instance, at 7 days and 6% cement, q_u_ rose from 693 kPa to 1.03 MPa (+49%), and *G*_o_ from 4.12 GPa to 4.92 GPa (+19%). After 28 days, both 15% and 30% CAR mixtures exhibited comparable strength levels (around 1.35–1.38 MPa), but the 30% CAR mixture showed more uniform densification and lower variability in results. The angular morphology and rough texture of CAR particles, observed in SEM images, facilitated better mechanical interlock with the soil and cementitious matrix, thus reducing porosity and enhancing load transfer capacity. These findings demonstrate that CAR can effectively act as a complementary stabilizing material, improving the compacted fabric and long-term performance of cemented soils.

The influence of compaction was evident across all specimens, highlighting the critical role of dry unit weight (*γ*_d_) in controlling the porosity and cementation efficiency of the stabilized soil. As the dry density increased from approximately 1.69 g/cm^3^ to 1.81 g/cm^3^, both q_u_ and *G*_o_ exhibited substantial improvements. The maximum recorded values— q_u_ = 1.55 MPa and *G*_o_ = 6.79 GPa—were obtained for the mixture containing 30% CAR and 6% cement at 1.81 g/cm^3^ after 28 days. Conversely, at 1.69 g/cm^3^, q_u_ and *G*_o_ decreased to 0.85 MPa and 4.82 GPa, representing reductions of 45% in strength and 30% in stiffness. These results demonstrate that denser compaction reduces initial porosity, enhances particle interlocking, and increases the number of cemented contacts per unit volume, which directly improves the mechanical performance of the soil–CAR–cement system. This behavior also validates the relevance of the porosity–binder index as a rational variable for normalizing the influence of compaction on mechanical response.

The relationship between the mechanical response and the porosity–binder index exhibited a strong power-law behavior, allowing accurate prediction of strength and stiffness over a wide range of porosities and cement contents. The best-fitting exponents were *x* = 0.27 for q_u_ and *x* = 0.38 for *G*_o_, indicating that the sensitivity of stiffness to binder content is greater than that of strength. These values are consistent with theoretical frameworks proposed by Diambra et al. [33] and Consoli et al. [34], who demonstrated that the exponent x reflects the balance between the cemented and uncemented phases within the composite material. A lower exponent (=0.27) implies that strength is more strongly governed by the reduction in porosity than by the volumetric cement content, emphasizing the dominance of physical interlocking and densification mechanisms in the load-bearing capacity. Conversely, the higher exponent for stiffness (0.38) suggests that small-strain behavior is more sensitive to the volume and continuity of cementitious bonds, which directly enhance elastic wave propagation through the solid skeleton. The parameter x is also inversely related to the empirical constant B, which links peak strength to the state parameter; in this study, the relationship *x* = 1.43/*B* was confirmed, consistent with the theoretical derivations for artificially cemented granular materials. Overall, decreasing $\eta /C_{iv}^{x}$ values corresponded to increasing q_u_ and *G*_o_, confirming that both properties improve as porosity decreases and binder concentration increases. Although CAR behaved as a mechanically inert component, its angular grains enhanced particle interlocking, further contributing to the observed strength gains. The measured q_u_ values ranged between 1.6–1.8 MPa at 7 days and 2.2–2.6 MPa at 28 days, corroborating the progressive hydration and microstructural densification of the cemented matrix over time.

A strong power-law relationship between the unconfined compressive strength (q_u_) and the porosity–binder index $\eta /C_{iv}^{x}$ was obtained for all stabilized mixtures, as shown in Equations (8)–(11). The determination coefficients (*R*^2^ = 0.85–0.98) confirmed the high predictive capacity of the index for cemented soil–CAR blends at both curing periods. The optimal exponent of 0.27 yielded the best fit, demonstrating that the interaction between porosity reduction and cement volume fraction primarily controls the q_u_. For the 7-day curing period, mixtures with 15% and 30% CAR achieved *R*^2^ values of 0.85 and 0.94, respectively, indicating that early-age strength is more affected by particle packing and compaction energy than by cement hydration. After 28 days, the same exponent remained valid, with the constants increasing to reflect strength gains over curing time; the scalar ratio between Equations (8) and (11) indicates a 37% increase in q_u_ from 7 to 28 days for 15% CAR, while the 30% CAR mixture reached the highest fit (*R*^2^ = 0.98) and predicted q_u_, emphasizing the synergistic effect of coarse aggregate residue on load transfer efficiency. These findings validate the porosity–binder index as a reliable normalization parameter for the mechanical performance of cemented fine–coarse mixtures, consistent with trends reported for other cemented geomaterials [12,16]. Comparable results have been reported by Consoli et al. [34], who demonstrated that porosity–binder relationships effectively normalize strength behavior in RAP–rock powder–cement mixtures. Their reported correlations (*R*^2^ = 0.95) align closely with the values obtained in this study, confirming that the *η*/C_iv_ framework remains robust across different geomaterials and stabilizer combinations.

(8) $$q_{u}=3.3394\times 10^{10}\;{\left[\frac{\eta }{{\left(C_{\mathrm{iv}}\right)}^{0.27}}\right]}^{-5.21}\;(R^{2}=0.85)\;15\%\mathrm{CAR}\mathrm{and}7\mathrm{days}$$

(9) $$q_{u}=4.3604\times 10^{10}\;{\left[\frac{\eta }{{\left(C_{\mathrm{iv}}\right)}^{0.27}}\right]}^{-5.21}(R^{2}=0.94)\;15\%\mathrm{CAR}\mathrm{and}7\mathrm{days}$$

(10) $$q_{u}=4.5657\times 10^{10}\;{\left[\frac{\eta }{{\left(C_{\mathrm{iv}}\right)}^{0.27}}\right]}^{-5.21}(R^{2}=0.97)\;15\%\mathrm{CAR}\mathrm{and}28\mathrm{days}$$

(11) $$q_{u}=5.6882\times 10^{10}\;{\left[\frac{\eta }{{\left(C_{\mathrm{iv}}\right)}^{0.27}}\right]}^{-5.21}(R^{2}=0.98)\;30\%\mathrm{CAR}\mathrm{and}28\mathrm{days}$$

A strong correlation was observed between the small-strain shear modulus (*G_o_*) and the porosity–binder index $\eta /C_{iv}^{x}$ for all tested mixtures, confirming the robustness of the power-law relationship described by Equations (12)–(15). Determination coefficients (*R*^2^ = 0.87–0.96 for 7 days and 0.91–0.98 for 28 days) demonstrate the high predictive capability of the index in capturing the stiffness evolution of cemented soil–CAR blends. The best-fitting exponent of 0.38 indicates that stiffness is more sensitive to binder content than q_u_, reflecting the dependency of elastic wave propagation on the continuity and rigidity of cementitious bonds. For the 7-day curing period, the equations for 15% and 30% CAR yielded *R*^2^ values of 0.88 and 0.95, respectively, showing that higher CAR content enhances the early stiffness due to improved granular interlocking. After 28 days, the scalar constants increased, signifying continued hydration and microstructural densification, with the 30% CAR mixture exhibiting the highest fit (*R*^2^ = 0.98) and predicted stiffness. The ratio between Equations (12) and (15) for 15% CAR reveals a 22% increase in *G*_o_ over the curing period, while the 30% CAR mixture showed an even higher growth rate, confirming the synergistic contribution of coarse particles to the bonded matrix. These findings validate the $\eta /C_{iv}^{0.38}$ index as a rational parameter for predicting small-strain stiffness in cemented fine–coarse systems, in agreement with the trends reported by Hoch et al. [35] and Bruschi et al. [36].

(12) $$G_{o}=2.8824\times 10^{7}\;{\left[\frac{\eta }{{\left(C_{\mathrm{iv}}\right)}^{0.38}}\right]}^{-2.69}(R^{2}=0.88)\;15\%\mathrm{CAR}\mathrm{and}7\mathrm{days}$$

(13) $$G_{o}=3.4716\times 10^{7}\;{\left[\frac{\eta }{{\left(C_{\mathrm{iv}}\right)}^{0.38}}\right]}^{-2.69}(R^{2}=0.95)\;30\%\mathrm{CAR}\mathrm{and}7\mathrm{days}$$

(14) $$G_{o}=3.5428\times 10^{7}\;{\left[\frac{\eta }{{\left(C_{\mathrm{iv}}\right)}^{0.38}}\right]}^{-2.69}(R^{2}=0.93)\;15\%\mathrm{CAR}\mathrm{and}28\mathrm{days}$$

(15) $$G_{o}=4.0826\times 10^{7}\;{\left[\frac{\eta }{{\left(C_{\mathrm{iv}}\right)}^{0.38}}\right]}^{-2.69}(R^{2}=0.98)\;30\%\mathrm{CAR}\mathrm{and}28\mathrm{days}$$

### 3.2. Effects of Porosity/Cement Index and Curing Periods on Stiffness for Soil−CAR−Cement Blends

An examination of Equations (8)–(11) and (12)–(15) reveals that the governing exponents remain identical across all formulations, whereas the scalar coefficients associated with q_u_ and *G*_o_ differ in magnitude. According to Consoli et al. [34], the mechanical response of soil–cement composites prepared with the same binder type can be standardized by incorporating controlled variables such as curing time and temperature. Hence, it becomes feasible to establish a unified mathematical framework capable of predicting the overall mechanical behavior of stabilized materials. The relationships defined in Equations (8)–(15) can therefore be normalized by dividing each by a reference strength (q_u_) or stiffness (*G*_o_) corresponding to a specific porosity–binder ratio, where $\eta /C_{iv}^{x}$= Δ. This normalization yields dimensionless forms of q_u_ (q_u-n_) and *G*_o_ (*G*_o-n_) for a given Δ, leading to the generalized predictive expressions presented in Equations (16) and (17).

(16) $$\frac{q_{u}}{q_{u-n}\left(\eta /C_{\mathrm{iv}}^{x}=\Delta \right)}=\frac{A{\left(\eta /C_{\mathrm{iv}}^{x}\right)}^{-B}}{A{\left(\Delta \right)}^{-B}}=\Delta^{B}{\left(\eta /C_{\mathrm{iv}}^{x}\right)}^{-B}$$

(17) $$\frac{Go}{Go-n\left(\eta /C_{\mathrm{iv}}^{x}=\Delta \right)}=\frac{A{\left(\eta /C_{\mathrm{iv}}^{x}\right)}^{-B}}{A{\left(\Delta \right)}^{-B}}={\;\Delta }^{B}{\left(\eta /C_{\mathrm{iv}}^{x}\right)}^{-B}$$

Equations (14) and (15) enable the assessment of the mechanical strength and stiffness of cemented soil mixtures across a wide range of porosities and binder contents using a single reference value for q_u_ and *G*_o_. When the parameter Δ = 30, these general expressions simplify to the specific predictive formulations presented in Equations (18) and (19).

(18) $$\frac{q_{u}}{q_{u-n}\left(\eta /C_{\mathrm{iv}}^{0.27}=30\%\right)}=49636567.024{\left(\eta /C_{\mathrm{iv}}^{0.27}\right)}^{-5.21}\left(R^{2},=,0.92\right)$$

(19) $$\frac{G_{o}}{G_{o-n}\left(\eta /C_{\mathrm{iv}}^{0.38}=30\%\right)}=9407.10{\left(\eta /C_{\mathrm{iv}}^{0.38}\right)}^{-2.69}\left(R^{2},=,0.99\right)$$

**Figure 12:**
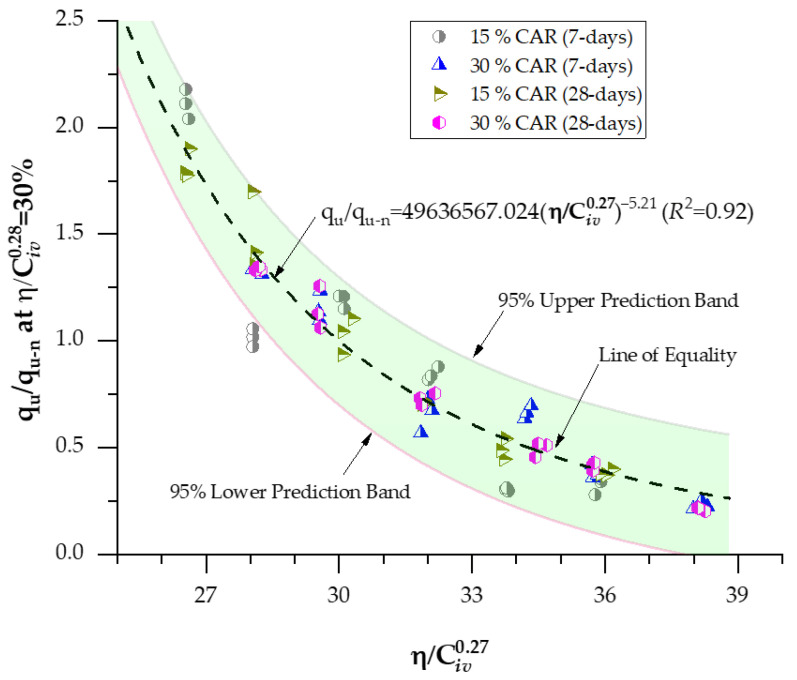
Normalization of the q_u_ at $\eta /C_{iv}^{0.28}=30$ considering 15–30% CAR and 7–28 days of curing.

**Figure 13:**
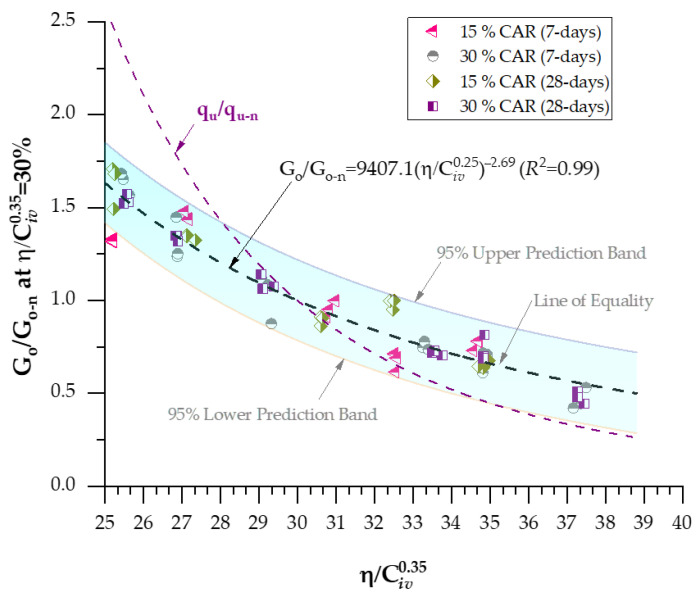
Normalization of stiffness at $\eta /C_{iv}^{0.28}=30$ considering 15–30% CAR and 7–28 days of curing.

The normalization of the mechanical results using Equations (18) and (19) yielded high determination coefficients (*R*^2^ = 0.92 for q_u_ and *R*^2^ = 0.99 for *G*_o_), confirming the strong predictive ability of the proposed model across varying curing periods and CAR contents. These results demonstrate that it is feasible to normalize the mechanical response of cemented soil–CAR mixtures through a single porosity–binder reference value ($\eta /C_{iv}^{x}\;$= 30%), effectively consolidating the influence of both porosity and binder content into a unified predictive expression. The elevated *R*^2^ values indicate the model’s robustness and its capacity to represent the strength and stiffness behavior of the mixtures with high fidelity. Figure 12 illustrates the normalized q_u_ behavior at $\eta /C_{iv}^{x}\;$= 30% for mixtures containing 15–30% CAR and cured for 7–28 days, while Figure 13 presents the corresponding normalized stiffness results at $\eta /C_{iv}^{x}\;$= 30%. The superposition of these normalized curves confirms the coherence of the proposed formulation, as both datasets converge onto a single master curve [30,35].

These trends are consistent with previous studies that reported mechanical improvements in soils stabilized with rock powders. For example, Blayi et al. [11] observed significant increases in strength and CBR when expansive soils were treated with silicon-rich rock powder, while Gidigasu et al. [12] also documented improvements in lateritic soils stabilized with crushed rock residues. The present CAR–cement mixtures exhibit similar strengthening mechanisms driven by densification and granular interlocking.

### 3.3. ML Application

**Figure 14:**
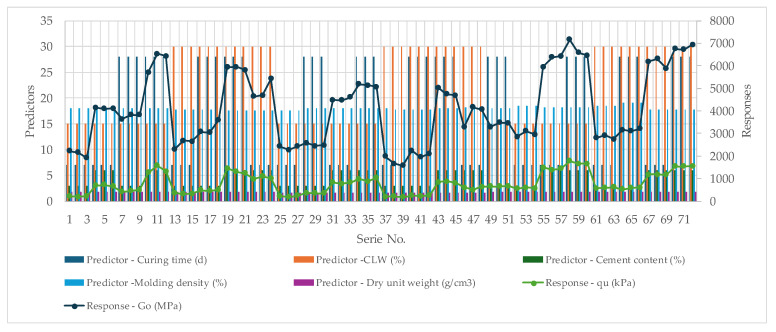
Identification of experimental measurements based on specific predictor variables for the *G_o_* and qu responses.

The use of cement content, dry unit weight, curing time, molding density, and CAR as input features represents a physically meaningful and data-informed approach to modeling the mechanical behavior of the material. These parameters have been employed as predictors in the machine learning framework developed to estimate the responses *G_o_* and *q_u_*. Each variable contributes differently to the material’s mechanical performance: higher cement content enhances bonding and strength gain; increased dry unit weight and molding density improve compactness and stiffness; longer curing times promote continued hydration and strength development; while CAR encapsulates compositional or microstructural characteristics influencing the overall response. A sensitivity analysis was conducted to quantify the relative influence of each predictor on *G_o_* and *q_u_*, thereby enhancing model interpretability and supporting future feature selection. Figure 14 presents the order of magnitude and distribution of the predictor variables, together with the corresponding ranges of the response values, providing a comprehensive overview of the dataset used for model development.

Table 4 summarizes the statistical results obtained from the 28 ML algorithms tested in this research. Among all models, the Exponential Gaussian Process Regression (GPR) exhibited the most accurate and robust performance for both responses. The statistical indicators confirmed its superior predictive capability in both the validation and testing phases. Specifically, for q_u_, RMSE and R^2^ values of 46.6 kPa and 0.988 were obtained during validation, and 155.0 kPa and 0.898 during testing. For *G_o_*, RMSE and R^2^ values of 284.8 MPa and 0.970 were achieved in validation, and 193.8 MPa and 0.975 in testing. This preset was selected on the basis of yielding the lowest RMSE and the R^2^ values closest to 1.0.

Overall, the GPR-based models—particularly those with exponential, Matern 5/2, and rational quadratic kernels—consistently outperformed other algorithms, achieving R^2^ values above 0.96 in most cases. Neural network architectures also demonstrated strong performance, though slightly less stable across testing stages. In contrast, coarse decision trees and kernel regression approaches yielded comparatively poor predictive accuracy, as evidenced by low or even negative R^2^ values. These findings indicate that nonlinear, kernel-based learning methods such as GPR effectively capture the complex relationships between the input features and the mechanical responses of the material. Consequently, the Exponential GPR model was selected as the optimal predictive framework for subsequent analyses.

This ML method provided the best performance compared with the traditional regression approach, as the validation stage yielded R^2^ values above 0.97, whereas the conventional method achieved a maximum value of 0.92. Considering the Exponential GPR model, the relationship between the predicted and true values was analyzed to evaluate the accuracy of the selected model.

**Figure 15:**
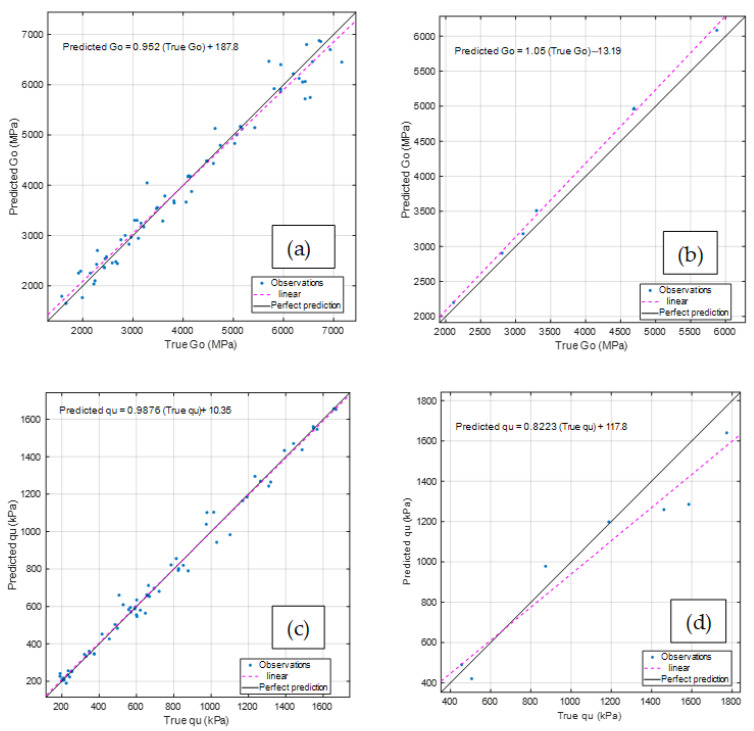
Comparison between predicted and true values: (**a**) validation stage for Go; (**b**) testing stage for Go; (**c**) validation stage for q_u_; and (**d**) testing stage for q_u_.

Moreover, the testing stage results confirmed that the model does not exhibit overfitting issues, as a strong agreement was also observed between predicted and true responses in this phase. Figure 15 illustrates the comparison between the predicted and measured values of *G_o_* and q_u_, based on the dataset of 75 collected measurements. The comparison shows that, during the validation stage, the linear trend closely aligns with the perfect prediction (represented by the 45° black line), while in the testing stage, only minor deviations from this ideal trend are observed. These results further validate the robustness and generalization capability of the selected ML framework.

**Figure 16:**
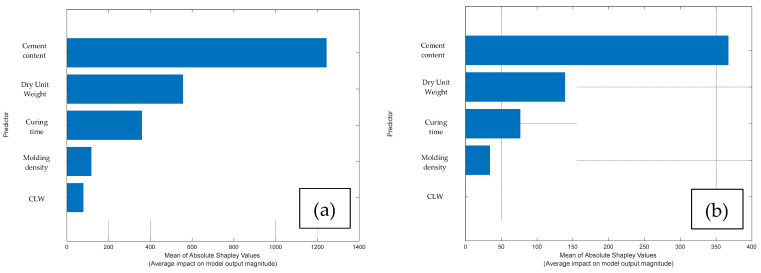
Computation of Shapley value for: (**a**) *Go*; and (**b**) q_u_.

To demonstrate the importance of the predictors in determining the *G_o_* and q_u_ values, the Shapley values were computed, as shown in Figure 16. In order of importance, cement content was identified as the most influential predictor, followed by dry unit weight, curing time, molding density, and finally CAR, which exhibited the lowest relative contribution. This order of importance was consistent for both responses.

Although the ML model in this study was based on five primary predictors, other factors may theoretically influence mechanical response and model accuracy. These include natural variability in moisture content and suction, curing temperature, cement fineness, soil mineralogy, and CAR particle morphology. While these variables were controlled to isolate the effects of mixture composition and compaction, their inclusion in future datasets would allow capturing additional nonlinear interactions and further improve the robustness of ML-based predictions.

### 3.4. Microstructural Analysis

**Figure 17:**
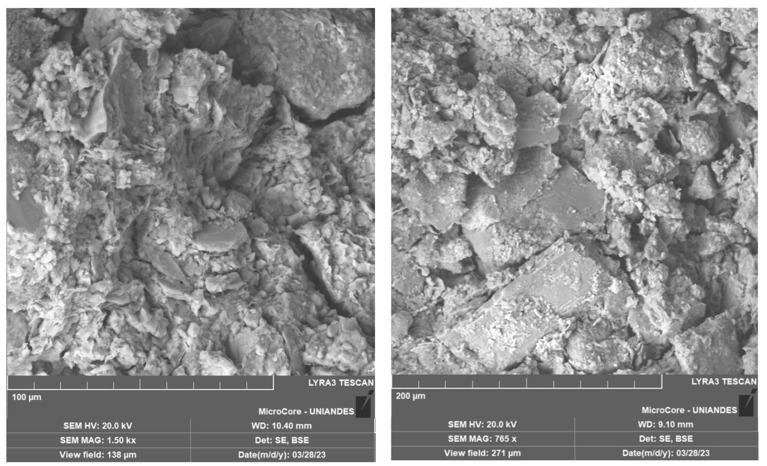
Microstructural analysis of SEM-EDX to soil-cement-CAR mixes.

**Figure 18:**
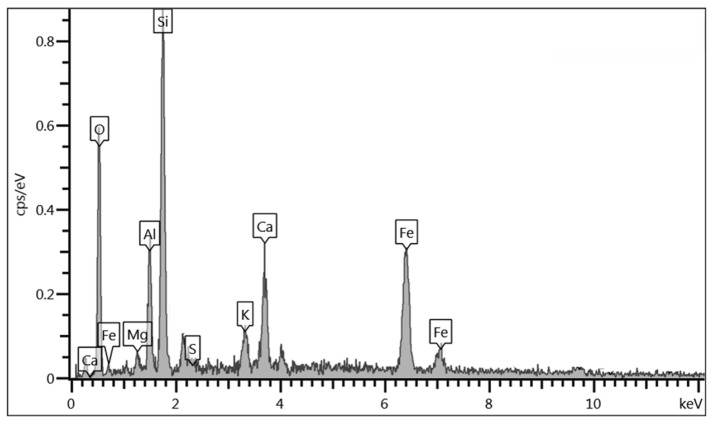
EDS analysis on soil-cement-CAR mixes.

Figure 17 presents the microstructure of two soil mixtures stabilized with crushed aggregate residue (CAR) and Portland cement, both cured for 28 days. Sample 1 exhibits a dense and compacted structure with visible microcracks between particles and the formation of C–S–H gels resulting from cement hydration. Similarly, Sample 2 shows coarse CAR particles that remained unreacted but were effectively embedded and cemented within the soil–cement interfaces. To further investigate these features, Figure 18 displays the EDX microanalysis performed on Sample 1. A high concentration of silica (Si) and alumina (Al), along with notable amounts of calcium (Ca) and iron (Fe), was detected at the analyzed interface. These chemical elements originate partly from the soil and the limestone residue (in the case of silica and alumina) and from the cement hydration products (C–S–H phases rich in calcium).

The presence of well-distributed C–S–H gels within the pore structure and along the particle interfaces indicates extensive cement hydration, which contributes to the enhanced bonding and densification of the mixtures. The compacted microstructure with reduced pore connectivity explains the significant increase in both q_u_ and *G_o_* at 28 days, as the solidified C–S–H matrix effectively transfers loads and restricts particle movement under stress. Additionally, the inclusion of CAR improved the internal skeleton of the material, serving as rigid inclusions that promoted mechanical interlock and reduced the total void ratio. The coexistence of unreacted CAR particles and cemented regions suggests that the stabilization mechanism is governed by both physical and chemical processes—particle interlocking and cementitious bonding—resulting in a dense, cohesive, and mechanically robust composite material. Similar microstructural observations were described by Blayi et al. [11], who reported the formation of cementitious gels and improved particle bonding in soils treated with silicon-rich powders. Likewise, the embedded and well-cemented CAR particles observed in our SEM images resemble the interlocking and densification mechanisms described in prior studies.

## 4. Conclusions

The mechanical performance of the stabilized mixtures was governed by the proportions of CAR and Portland cement, together with compaction level and curing time. Mixtures with higher CAR and cement contents achieved the greatest gains in strength and stiffness, with the 30% CAR–6% cement blends showing the best overall performance after curing.

Curing from 7 to 28 days and increasing dry unit weight notably enhanced q_u_ and *G*_o_, confirming the progressive development of cementation and matrix densification. Compacted mixtures at higher densities consistently exhibited stiffer and stronger behavior.

The porosity–binder index ($\eta /C_{iv}^{x}$) successfully normalized the combined effects of compaction, binder dosage, and cement content, producing strong correlations for both q_u_ (*R*^2^ = 0.94) and *G*_o_ (*R*^2^ = 0.92). These results align with previous studies using porosity–binder frameworks for cemented geomaterials.

SEM–EDX observations revealed the formation of C–S–H and C–A–S–H gels and the effective embedding of CAR particles within the soil–cement matrix, supporting the mechanical improvements observed at later curing ages.

Machine learning models, particularly those based on Gaussian Process Regression, provided superior predictive accuracy (*R*^2^ > 0.97) compared with the empirical index alone, demonstrating their capacity to capture nonlinear interactions among mixture variables.

Incorporating CAR into soil–cement mixtures proved technically viable and environmentally beneficial. CAR enhanced mechanical performance while enabling the reuse of quarry by-products and contributing to reduced cement consumption. The combined η/C_iv_–ML framework offers a robust basis for optimizing mixture design in sustainable subgrade and foundation applications.

## Figures and Tables

**Table 1:** Influence of various types of rock aggregate on soil improvement and new geomaterials development.

Type of Powder Rock	Type of Soil/Residue	Binder	*w* (%)	Molding *γ*_d_ (kN.m^−3^)	Strength (MPa)	Ref.
Volcanic rock dacite (30%)	Reclaimed asphalt pavement (70%)	Cement (3%)	8	20.0	0.667	[10]
Volcanic rock dacite (30%)	Reclaimed asphalt pavement (70%)	Cement (3%)	8	21.0	1.385	[10]
Volcanic rock dacite (30%)	Reclaimed asphalt pavement (70%)	Cement (3%)	8	22.0	2.350	[10]
Silicon natural rock powder (8%)	Expansive soil (MH)	-	16.75	18.35	0.2435	[11]
+Silicon natural rock powder (16%)	Expansive soil (MH)	-	16.00	18.52	0.3036	[11]
Silicon natural rock powder (24%)	Expansive soil (MH)	-	16.75	18.35	0.363	[11]
Granitic powder rock	Lateritic soil	Carbide Lime (3%)	10.05	20.40	89.99% (*CBR*)	[12]
Granitic powder rock	Lateritic soil	Carbide Lime (5%)	10.25	20.70	168.53% (*CBR*)	[12]
Granitic powder rock	Lateritic soil	Carbide Lime (7%)	10.45	21.10	185.64% (*CBR*)	[12]
Granitic powder rock (30%)	-	Cement (3%) and PPF (0.5%)	9.0	20.0	1200 (*Mr*)	[13]

MH: high-plasticity silt. PPF: polypropylene fiber. CBR: California Bearing Ratio. Mr: Resilient Modulus.

**Table 2:** Physical properties of the soil sample and CAR. (*D* is the particle diameter) [26].

Properties	Soil	CAR
LL Limit Liquid of soil, %	42.00	-
PL Plastic Limit of soil, %	26.05	-
PI Plastic Index of soil, %, (i.e., LL-PL)	15.95	-
Gravel particles (*D*-2 mm), %	0	41
Coarse sand particle size (0.6 mm- *D*-2 mm), %	0	32
Medium sand particle size (0.2 mm- *D*-0.6 mm), %	0	13
Fine sand particle size (0.06 mm- *D*-0.2 mm), %	12	14
Silt particle size (0.002 mm- *D*-0.06 mm), %	78	-
Clay particles size (*D* < 0.002 mm), %	10	-
Effective diameter (D_10_), mm	0.0021	0.15
Mean particle diameter (D_50_), mm	0.011	1.6
Uniformity coefficient of materials (C_u_)	7.14	13.67
Coefficient of curvature of materials (C_c_)	0.96	1.59
The specific gravity of the soil sample and CAR	2.80	2.52
Activity of clay, A [A = PI/(% < 0.002 mm)]	1.60	-
Color of raw materials	Black	Gray
Classification of raw materials (USCS)	CL	SW

**Table 3:** Chemical composition of the soil sample and CAR [26].

Element	Soil Composition (%)	CAR Composition (%)
SiO_2_	66	9.0
Al_2_O_3_	21.7	1.3
SO_3_	5.0	-
K_2_O	3.1	-
CaO	3.0	72.4
Fe_2_O_3_	0.9	0.9
TiO_2_	0.3	-
MgO	-	2.1
Mn	-	14.3

**Table 4:** Statistical measures of machine learning presets.

Preset	q_u_				*G_o_*			
	Validation		Testing		Validation		Testing	
	RMSE (kPa)	R^2^	RMSE (kPa)	R^2^	RMSE (MPa)	R^2^	RMSE (MPa)	R^2^
Fine Tree	170.6	0.845	295.2	0.629	534.6	0.894	231.9	0.964
Linear	132.9	0.906	200.5	0.829	417.5	0.936	380.5	0.903
Interactions Linear	89.1	0.958	156.6	0.896	397.7	0.942	243.2	0.960
Robust Linear	135.0	0.903	192.0	0.843	425.8	0.933	390.2	0.898
Stepwise Linear	113.8	0.931	155.5	0.897	428.4	0.932	380.5	0.903
Medium Tree	220.3	0.742	393.3	0.341	688.5	0.825	290.6	0.943
Coarse Tree	433.7	0.000	621.6	−0.646	1645.7	0.000	1264.2	−0.070
Linear SVM	140.9	0.894	189.8	0.847	424.2	0.934	407.8	0.889
Quadratic SVM	83.9	0.963	171.6	0.875	436.8	0.930	312.2	0.935
Cubic SVM	254.2	0.656	140.1	0.916	2151.4	−0.709	208.8	0.971
Fine Gaussian SVM	171.7	0.843	174.7	0.870	512.4	0.903	229.5	0.965
Medium Gaussian SVM	90.0	0.957	164.0	0.886	359.9	0.952	225.6	0.966
Coarse Gaussian SVM	193.6	0.801	293.6	0.633	564.7	0.882	377.7	0.905
Efficient Linear Least Squares	261.3	0.637	299.9	0.617	788.1	0.771	729.3	0.644
Efficient Linear SVM	372.9	0.261	544.4	−0.262	1575.5	0.084	1365.0	−0.247
Boosted Trees	127.3	0.914	302.7	0.610	460.4	0.922	175.9	0.979
Bagged Trees	155.6	0.871	340.1	0.507	584.7	0.874	273.3	0.950
Squared Exponential GPR	49.9	0.987	150.6	0.903	301.2	0.966	214.0	0.969
Matern 5/2 GPR	47.9	0.988	150.8	0.903	294.0	0.968	209.4	0.971
Exponential GPR	46.6	0.988	155.0	0.898	284.8	0.970	193.8	0.975
Rational Quadratic GPR	47.3	0.988	150.7	0.903	291.2	0.969	208.4	0.971
Narrow Neural Network	104.1	0.942	155.3	0.897	327.0	0.961	179.7	0.978
Medium Neural Network	71.6	0.973	150.5	0.903	1738.0	−0.115	186.6	0.977
Wide Neural Network	93.0	0.954	151.4	0.902	1357.7	0.319	224.1	0.966
Bilayered Neural Network	67.0	0.976	152.2	0.901	761.0	0.786	205.7	0.972
Trilayered Neural Network	65.2	0.977	160.6	0.890	662.8	0.838	200.5	0.973
SVM Kernel	440.4	−0.032	693.3	−1.047	1678.9	−0.041	1222.9	−0.001
Least Squares Regression Kernel	210.9	0.764	323.7	0.554	738.0	0.799	456.4	0.861

## Data Availability

The original contributions presented in the study are included in the article, further inquiries can be directed to the corresponding authors.
